# Peripheral Neuropathy Secondary to Ertapenem Administration for Treatment of Infectious Endocarditis

**DOI:** 10.7759/cureus.37253

**Published:** 2023-04-07

**Authors:** Patrick J Buckman, Carly Scatton, James J Cappola

**Affiliations:** 1 Internal Medicine, Campbell University School of Osteopathic Medicine, Lillington, USA; 2 Neurology, Doylestown Health, Doylestown, USA

**Keywords:** chronic kidney disease, neurotoxicity, invanz, drug reaction, peripheral neuropathy, ertapenem

## Abstract

Ertapenem is a widely used broad-spectrum carbapenem antibiotic active against most species of gram-negative and gram-positive aerobes and anaerobes with specific targeting of the Amp C extended-spectrum beta-lactamases. It is advantageous for its once-daily dosing via IM or IV administration and minimal side effect profile for the treatment of community-acquired infections.

We report an 80-year-old man presenting with reversible peripheral neuropathy following the administration and subsequent discontinuation of ertapenem for the treatment of acute infectious endocarditis. It is especially notable that our patient had less severe (i.e., stage 3) chronic kidney disease as opposed to current literature which only presents similar findings with more advanced kidney disease (stage 4-5). Upon cessation of ertapenem administration, our patient recovered to his baseline motor and sensory status over a period of four days. We believe there to be value in reporting this case, as well as value in reevaluating the current recommendations for ertapenem dosing in those with kidney compromise.

## Introduction

While there are some rare cases of seizures or encephalopathy following the administration of ertapenem [1], there is only one other study documenting cases of peripheral neuropathy following its administration - the patients in which peripheral neuropathy occurred had stage 4 or stage 5 chronic kidney disease versus stage 3 in our patient [2]. In addition, these previously reported patients developed sensory deficits but no motor deficits. Ertapenem is primarily cleared through renal metabolism, with direct renal clearance constituting up to 50% of the clearance and the majority of the rest being through a beta-lactam metabolite that is excreted in urine [3].

Peripheral neuropathy is a term that refers to a group of dysfunctions related to peripheral neuronal abnormalities commonly associated with sensory loss or weakness which may progress to abnormal gait and increased fall risk. The most common cause of peripheral neuropathy is diabetes mellitus. Peripheral neuropathy can present differently depending on the size of the nerve being impacted. Larger nerves tend to present with loss of balance and sensation while smaller nerves tend to present with more painful sensations such as prickling or burning [4].

We conducted an extensive literature review of multiple databases in March of 2022 for any other reported cases of ertapenem-related peripheral neuropathy or other nervous system abnormalities attributed to ertapenem. As of March 2022, there is only one other reported study of peripheral neuropathy secondary to ertapenem toxicity, detailing three patients with acute renal failure and use of renally-dosed ertapenem of 500 mg IV daily. One patient exhibited central nervous system deficits while the other two exhibited purely peripheral nervous system impairments. All three of these patients recovered normal neurological function within eight days following cessation of ertapenem. All three patients in this case report had chronic kidney stage 4 or 5, in contrast to our patient who had chronic kidney disease stage 3 [2].

There is an additional related case study detailing the effects of ertapenem on the nervous system with clinical manifestations including hallucinations, agitation, and delirium. Following cessation of ertapenem, the patient, in this case report, returned to baseline neurologic function [1]. There is one published article describing a patient with normal renal function who developed encephalopathy while receiving ertapenem. This patient’s encephalopathy resolved within two days [5].

There have been additional studies calling for a reevaluation of the current guidelines to safely dose ertapenem in patients with end-stage renal disease figure who are undergoing dialysis [6]. This case study presents an elderly patient who developed reversible peripheral neuropathy following ertapenem administration in the setting of stage 3 chronic kidney disease.

## Case presentation

We present an 80-year-old man with recurrent endocarditis secondary to poor dental health, atrial fibrillation, coronary artery disease, congestive heart failure, hyperlipidemia, and hypertension, as well as a history of stage 3b chronic kidney disease (GFR of 36). He presented to the emergency department with worsening shortness of breath, which started two to three weeks prior to admission following cardiac catheterization. This shortness of breath was originally attributed to severe fatigue secondary to anemia and aortic stenosis with mitral regurgitation.

At the time of the emergency department presentation, the patient’s creatinine was 1.8 mg/dL, hemoglobin was 9.2 g/dL, and hematocrit was 28.8%. Full emergency department laboratory results can be found in Table 1.

**Table 1:** Emergency department laboratory results obtained in the emergency department following a chief complaint of shortness of breath. Most notable are the increased creatinine values indicating decreased GFR as well as the decreased Hgb and HCT values. Worth noting is that the patient was anticoagulated.

Laboratory Test	Patient's Result	Reference Range
RBC	3.25 10^6 / uL	4.70-6.10
Hgb	9.2 g/dL	13.0-18.0
Hct	28.80%	39.0-52.0
MCHC	31.9 g/dL	22.0-37.0
RDW	20.30%	11.5-14.5
MPV	11.8 fL	7.4-10.4
Absolute Neuts (auto)	6.6 10^3/uL	1.4-6.5
Absolute Lymphs (auto)	0.6 10^3/uL	1.2-3.4
Neutrophils %	83.40%	42.2-75.2
Lymphocytes %	7.90%	20.5-51.1
PT	26.9 sec	11.4-14.6
Sodium	147 mmol/L	135-145
Chloride	110 mmol/L	98-107
BUN	47 mg/dL	9-20
Creatinine	1.8 mg/dL	0.7-1.3
Glucose	113 mg/dL	65-99
Total Bilirubin	1.8 mg/dL	0.2-1.3

Given his presenting symptoms of severe weakness and a hemoglobin of 9.2, the patient was administered two units of packed red blood cells to alleviate the anemia. A chest x-ray performed in the emergency department was determined to be unremarkable. The patient was admitted to inpatient services and was diagnosed with another case of infectious endocarditis. The patient had a history of poor dental hygiene which is likely the source of the infection, and he was on chronic penicillin to manage the risk of recurrent endocarditis. Due to the chronic penicillin, he was started on ertapenem 750 mg IV daily.

The patient was discharged after a two-week hospital stay with a plan to continue ertapenem 750 mg IV daily for a total course of six weeks. At baseline, the patient described chronic decreased sensation in his feet. Throughout the outpatient ertapenem infusions, the patient reported worsening fatigue, weakness, and sensory loss. After about three weeks of ertapenem, the patient’s weakness and sensorimotor deficits advanced to a point at which he could no longer ambulate even with assistive devices such as a rolling walker and crutches. His ability to complete normal activities of daily living was extremely limited.

The patient originally thought his weakness and loss of sensation were secondary to deconditioning. Over two weeks after discharge, the patient underwent outpatient physical therapy to help regain strength and to prepare him for a surgical mitral valve replacement. His physical therapist noted weakness in all four extremities during bilateral manual muscle testing, the report of which can be seen in Table 2.

**Table 2:** Physical therapy reporting of weakness following two weeks of daily ertapenem administration. Results are from manual muscle testing.

Motion Tested	Right	Left
Hip flexion	4/5	4+/5
Hip abduction	4-/5	4-/5
Knee flexion	4/5	4/5
Knee extension	4+/5	4+/5
Ankle dorsiflexion	4+/5	4+/5
Ankle plantar flexion	4/5	4/5

His physical therapist also reported decreased endurance, gait dysfunction, and increased fall risk. After two weeks of physical therapy, ertapenem was stopped due to concern for medication-induced neuropathy. Three to four days later, his lower extremity strength improved, and he again became ambulatory, returning to his baseline with no residual sensory deficits outside of his baseline sensory neuropathy. After the patient fully recovered clinically, an outpatient EMG was conducted which showed a return to the patient’s reported baseline neuropathy levels including mildly reduced sensation in the bilateral lower extremities.

## Discussion

Carbapenems act via inhibition of bacterial cell wall synthesis by inhibiting the function of penicillin-binding proteins. All carbapenems have about the same antibacterial coverage, though ertapenem does not cover some species of *Pseudomonas aeruginosa* and *Enterococcus* [7]. However, an advantage of ertapenem is its long half-life of four hours, which allows once-daily dosing. Once-daily dosing is ideal for outpatient infusion as with our patient [8]. The long half-life of ertapenem is primarily due to the fact that between 92 and 95% of the drug is protein-bound [9].

The most common causes of motor-predominant peripheral neuropathy are diabetes mellitus or toxins. Cryptogenic polyneuropathy is commonly seen in patients at high risk for prediabetes and metabolic syndrome. Peripheral neuropathy can present differently depending on the size of the nerve being impacted and the myelination diameter. Patients with damage to larger-diameter nerves tend to present with loss of balance and sensation while patients with damage to smaller-diameter nerves tend to present with more painful sensations such as prickling or burning.

Acute peripheral neuropathy is often caused by toxins or infectious agents. For example, motor-predominant presentations are typically secondary to lead intoxication, acute porphyria, or Kennedy disease [4]. Our patient demonstrates a typical presentation of neuropathy following drug toxicity as exhibited by the acute onset and motor-predominant effects.

A variety of chemotherapy drugs, especially platinum-based alkylating agents such as cisplatin may cause peripheral neuropathy [10]. The exact mechanism of the neurotoxicity, specifically peripheral neuropathy, caused by cisplatin and other alkylating agents is unknown. It is hypothesized that cisplatin directly inhibits the actions of fast-action transport in neuronal cells, although why this causes peripheral neuropathy is not well understood [11].

Lead-induced peripheral neuropathy is most commonly caused by industrial exposure but the exact mechanism is also unknown. One hypothesis is that lead impacts the blood-nerve barrier, inducing edema in Schwann cells and inhibiting their action [12]. Another hypothesis is that lead induces neuropathy through a mechanism similar to porphyric neuropathy caused by abnormal porphyrin metabolism [13]. Peripheral neuropathy from lead most commonly presents with weakness of the wrist and finger extensors. Lead toxicity can also cause a sensory loss in the lower extremities, predominantly in the feet, but lower extremity weakness has also been reported [14].

Diabetic peripheral neuropathy is a neurodegenerative disorder that targets the sensory and motor axons of the peripheral nervous system. The mechanisms are not entirely understood but involve the retraction of terminal sensory axons in the periphery with the preservation of the cell bodies. Due to its typical presentation, it is assumed that the longest sensory axons are targeted first [15]. These mechanisms are due to an overabundance of acylcarnitines, which are toxic to peripheral Schwann cells and dorsal root ganglion neurons. Acylcarnitines accumulate due to saturation of the axonal transport systems in patients with diabetes and subsequent failure to remove acetyl-CoA as a product of metabolism [16]. Additionally, excess substrate loads in patients with diabetes cause oxidative phosphorylation to fail, leading to increased reactive oxygen species (ROS) which also damage Schwann cells and dorsal root ganglion neurons [17]. Both of these mechanisms contribute to the peripheral neuropathy seen in diabetic patients.

Genetic conditions such as Charcot-Marie-Tooth (CMT) disease cause peripheral neuropathy. The classic presentation of CMT is gradual distal weakness and sensory loss in the first two decades of life. Any length-dependent neuropathy can potentially have a genetic component and CMT presents with gene mutations over 90% of the time in the *PMP22*, *GJB1*, *MPZ*, and *MFN2* genes [18]. An inherited neuropathy could explain our patient’s baseline peripheral neuropathy, although due to his advanced age, it is unlikely to be the primary cause.

Guillain-Barré syndrome (GBS) is also a well-known cause of peripheral neuropathy which affects peripheral nerves in both upper and lower extremities following a recent infection, for example, influenza virus or *Campylobacter jejuni *[19]. Vitamin B12 is a required vitamin for the maintenance and function of nervous system tissues, particularly in the periphery. A deficiency in vitamin B12 can lead to peripheral neuropathy [20].

It is not currently known why ertapenem causes peripheral neuropathy. Based on the different mechanisms listed above, the ertapenem-associated peripheral neuropathy seen in our patient most closely follows the presentation of patients with cisplatin-induced peripheral neuropathy. It is unlikely that our patient’s presentation was caused by an inherited syndrome such as CMT due to the patient’s advanced age relative to the typical onset of CMT symptoms. Likewise, due to the pure lower extremity presentation of our patient’s neuropathy, it would be unlikely to be secondary to GBS which typically presents in both upper and lower extremities simultaneously.

From the published literature on peripheral neuropathy secondary to ertapenem toxicity, male patients over the age of 65 with some level of kidney compromise seem to be at the highest risk of neurotoxicity. Decreased protein binding of the ertapenem in patients with decreased kidney function could explain the development of peripheral neuropathy in these patients. We propose that the current guidelines for ertapenem use in these high-risk patients be reevaluated.

## Conclusions

Ertapenem-induced peripheral neuropathy is a poorly understood and poorly reported secondary side effect of the drug when used in patients with kidney impairment. Current literature is limited on the extent and prevalence of ertapenem-induced peripheral neuropathy and other neurological impairments, and the established side effect profile is limited in regard to these neurological effects. The current guidelines for use of ertapenem in patients with kidney compromise may need to be revised to reflect these documented findings and to ensure the safety of future use of this drug in these patient populations.
